# Dose-effect relationship and molecular mechanism by which BMSC-derived exosomes promote peripheral nerve regeneration after crush injury

**DOI:** 10.1186/s13287-020-01872-8

**Published:** 2020-08-18

**Authors:** Jiuhong Zhao, Yali Ding, Rui He, Kui Huang, Lu Liu, Chaona Jiang, Zhuozhou Liu, Yuanlan Wang, Xiaokai Yan, Fuyang Cao, Xueying Huang, Yanan Peng, Rui Ren, Yuebin He, Tianwei Cui, Quanpeng Zhang, Xianfang Zhang, Qibing Liu, Yunqing Li, Zhijian Ma, Xinan Yi

**Affiliations:** 1 Key Laboratory of Brain Science Research & Transformation in Tropical Environment of Hainan Province, Hainan Medical University, Haikou, China; 2 Department of Anatomy, Hainan Medical University, Haikou, China; 3 School of Medicine, Tibet University, Lhasa, China; 4 Northwest Institute of Plateau Biology, Chinese Academy of Sciences, Xining, China

**Keywords:** Mesenchymal stem cells, Exosome, Neurons, Regeneration

## Abstract

**Background:**

The development of new treatment strategies to improve peripheral nerve repair after injury, especially those that accelerate axonal nerve regeneration, is very important. The aim of this study is to elucidate the molecular mechanisms of how bone marrow stromal cell (BMSC)-derived exosomes (EXOs) participate in peripheral nerve regeneration and whether the regenerative effect of EXOs is correlated with dose.

**Method:**

BMSCs were transfected with or without an siRNA targeting Ago2 (SiAgo2). EXOs extracted from the BMSCs were administered to dorsal root ganglion (DRG) neurons in vitro. After 48 h of culture, the neurite length was measured. Moreover, EXOs at four different doses were injected into the gastrocnemius muscles of rats with sciatic nerve crush injury. The sciatic nerve functional index (SFI) and latency of thermal pain (LTP) of the hind leg sciatic nerve were measured before the operation and at 7, 14, 21, and 28 days after the operation. Then, the number and diameter of the regenerated fibers in the injured distal sciatic nerve were quantified. Seven genes associated with nerve regeneration were investigated by qRT-PCR in DRG neurons extracted from rats 7 days after the sciatic nerve crush.

**Results:**

We showed that after 48 h of culture, the mean number of neurites and the length of cultured DRG neurons in the SiAgo2-BMSC-EXO and SiAgo2-BMSC groups were smaller than that in the untreated and siRNA control groups. The average number and diameter of regenerated axons, LTP, and SFI in the group with 0.9 × 10^10^ particles/ml EXOs were better than those in other groups, while the group that received a minimum EXO dose (0.4 × 10^10^ particles/ml) was not significantly different from the PBS group. The expression of PMP22, VEGFA, NGFr, and S100b in DRGs from the EXO-treated group was significantly higher than that in the PBS control group. No significant difference was observed in the expression of HGF and Akt1 among the groups.

**Conclusions:**

These results showed that BMSC-derived EXOs can promote the regeneration of peripheral nerves and that the mechanism may involve miRNA-mediated regulation of regeneration-related genes, such as VEGFA. Finally, a dose-effect relationship between EXO treatment and nerve regeneration was shown.

## Background

Clinical therapies for peripheral nerve injury still require improvement [1, 2]. The development of new treatment strategies to improve peripheral nerve repair after injury, especially those that accelerate axonal nerve regeneration, is very important [1–4]. Moreover, the application of biotherapy to motor and dorsal root ganglion (DRG) neurons remains a major obstacle in the treatment of various neurological conditions, including peripheral nerve injury, motor neuron disease, and pain. Since the neuronal soma plays an important role in the initiation and control of axonal regeneration, targeted administration is an attractive strategy for peripheral nerve injury [5]. Selective regulation of sensory neuron gene expression has many applications in the peripheral nervous system. Subcutaneous peripheral injection of plasmid DNA combined with a nonviral cationic gelatin (CG) vector (CG/DNA) induced the expression of DRG genes in rat lumbar DRGs [6]. CG/DNA undergoes rapid retrograde transport through the sciatic and spinal nerves, and reporter mRNA expression in L4 and L5 DRGs can be detected within 60 h [6]. Mesenchymal stem cells (MSCs) are self-proliferating multipotent stromal cells from the bone marrow, fat, dental pulp, umbilical cord blood, and other tissues [7–10]. It has been shown that BMSCs can promote regeneration [11–15], and on this basis, a paracrine-mediated mechanism has been established, and an increasing amount of evidence indicates that MSCs may provide a benefit due to their close proximity to damaged tissues through secretion of exosomes (EXOs), a type of extracellular vesicle (EV) [16–19]. The first data describing the ability of vesicles to function in intercellular communication were published in two very different papers in 1983. The term for these vesicles was coined by Johnstone et al. in 1989 [20, 21]. It is difficult to strictly classify all EVs, but until now, EVs were thought to mediate endocytosis when cells release multivesicular bodies [18, 19]. EVs range in size from 30 to 150 nm [21]. Proteomic analysis of BMSC-derived EXO contents showed the presence of many factors in conditioned medium from BMSCs [22]. EXO mRNAs and microRNAs (miRNAs) (together with proteins), which are both functional, fuse with the cell membrane when delivered to another cell; this leads to the translation of new proteins [2]. The intracellular transfer of EXOs has now been demonstrated in many different types of cells, and EXOs from all these cell types have demonstrated the ability to use delivered miRNAs [23, 24]. The characteristics of EXO uptake indicate that EXOs from donor cells shuttle through intracellular vesicles to the endoplasmic reticulum and lysosome during transport [24]. BMSCs secrete EXOs that contain more than 150 different miRNA molecules that can be transferred to target cells [25]. Various studies have shown that EXOs play a major role in the therapeutic effects of BMSCs. BMSC EXOs from humans, rats, and mice exert therapeutic effects, which are mediated by EXO protein and RNA cargo, in various nervous system injury models [14, 15]. More specifically, miRNAs carried by EXOs are involved in various key processes, such as nerve and vascular regeneration, and EXOs derived from Schwann cells, macrophages, and MSCs can promote peripheral nerve regeneration [24–27]. Vesna Bucan et al. [26] demonstrated that EXOs could promote the regeneration of injured nerves and improve motor functional recovery in regenerated nerves in a rat sciatic nerve crush (SNC) model. B Mead et al. [27] isolated EXOs from BMSCs and applied them to a rat optic nerve crush model, in which they promoted the survival of retinal ganglion cells (RGCs) and the regeneration of RGC axons while partially preventing RGC axonal loss and dysfunction. BMSC-derived EXOs demonstrated significant neuroprotective and neuritogenic effects when used to treat primary retinal cultures [27]. However, whether this effect on nerve regeneration is related to the concentration of EXOs has not yet been reported. In our previous study, we found that 5 days after EXOs were injected into the gastrocnemius muscle, significantly more EXOs in the DRG and spinal cord anterior horn were aggregated on the injection side than on the contralateral side, which indicates that EXOs undergo both retrograde axoplasmic transport and hematogenous transport (data not reported). Therefore, in this study, we injected different doses of EXOs into the gastrocnemius muscle in rats subjected to SNC to observe their nerve regenerative effects and preliminarily clarified the mechanism of this effect through molecular biological methods.

## Methods

### Animals

Fifty 6- to 8-week-old male and female Sprague-Dawley (SD) rats weighing 200–250 g (Changsha Tianqin Biotechnology, Changsha, Hunan, China) were housed in standard rat cages at a temperature of 21 °C and a humidity of 55% under a 12 h light-dark cycle. The rats had unlimited access to water and food and were cared for by well-trained personnel. Animal sacrifice and tissue collection were performed according to the guidelines of the China Laboratory Animal Nursing and Use Regulations and with the approval of the Animal Ethics Committee of Hainan Medical University.

### BMSC culture and transfection with siRNA

Primary BMSCs were harvested from the marrow of the femur and tibia of 2 SD rats, as previously described [27]. For cell culture, a total of 2 × 10^6^ BMSCs were initially seeded into each T75 flask (Corning, Acton, MA) and cultured in medium that contained 89% Minimum Essential Medium Eagle-α-modification (α-MEM) (Gibco, 12561-056, Gaithersburg, MD, USA), 10% EXO-depleted FBS (Gibco, 10099141), and 1% penicillin/streptomycin (HyClone, SV30010, Logan, UT, USA). The cells were maintained at 37 °C in 5% CO_2_, and the cell culture medium was changed every 72 h. Efforts were made to passage the cells when they reached 80% confluence; for this, 0.05% trypsin/EDTA (Thermo Fisher Scientific, 25300054, Grand Island, USA) was used. The passage 6 cells were analyzed by fluorescence-activated cell sorting (FACS) (ACEA, NovoCyte 1040 cytometer) using antibodies against both the CD45 (FITC-conjugated anti-rat CD45, eBioscience, 11-0461-80, Vienna, Austria) and CD44 (PE-conjugated anti-rat CD44, eBioscience, 12-0444-80) biomarkers [28]. To transfect the BMSCs with siRNA, Lipofectamine 3000 (Thermo Fisher, L3000008) was used according to the vendor’s protocol. Briefly, passage 6 BMSCs grown to 70% confluence in α-MEM were incubated with Lipofectamine 3000 reagent and an siRNA targeting Ago2 (SiAgo2) (Sino Biological, 3263, Peking, PRC) or an interference control siRNA (SiScr) for 48 h.

### Cell viability assay

BMSCs were seeded on 96-well plates at a density of 5000 cells/well and incubated in serum-free medium for 72 h. Then, the MTS Cell Proliferation Assay was applied and the plates were read by a spectrometer. The viability was determined by normalizing BMSCs cultured in serum-free medium to those cultured in 10% FBS.

### EXO isolation

**Fig. 1:**
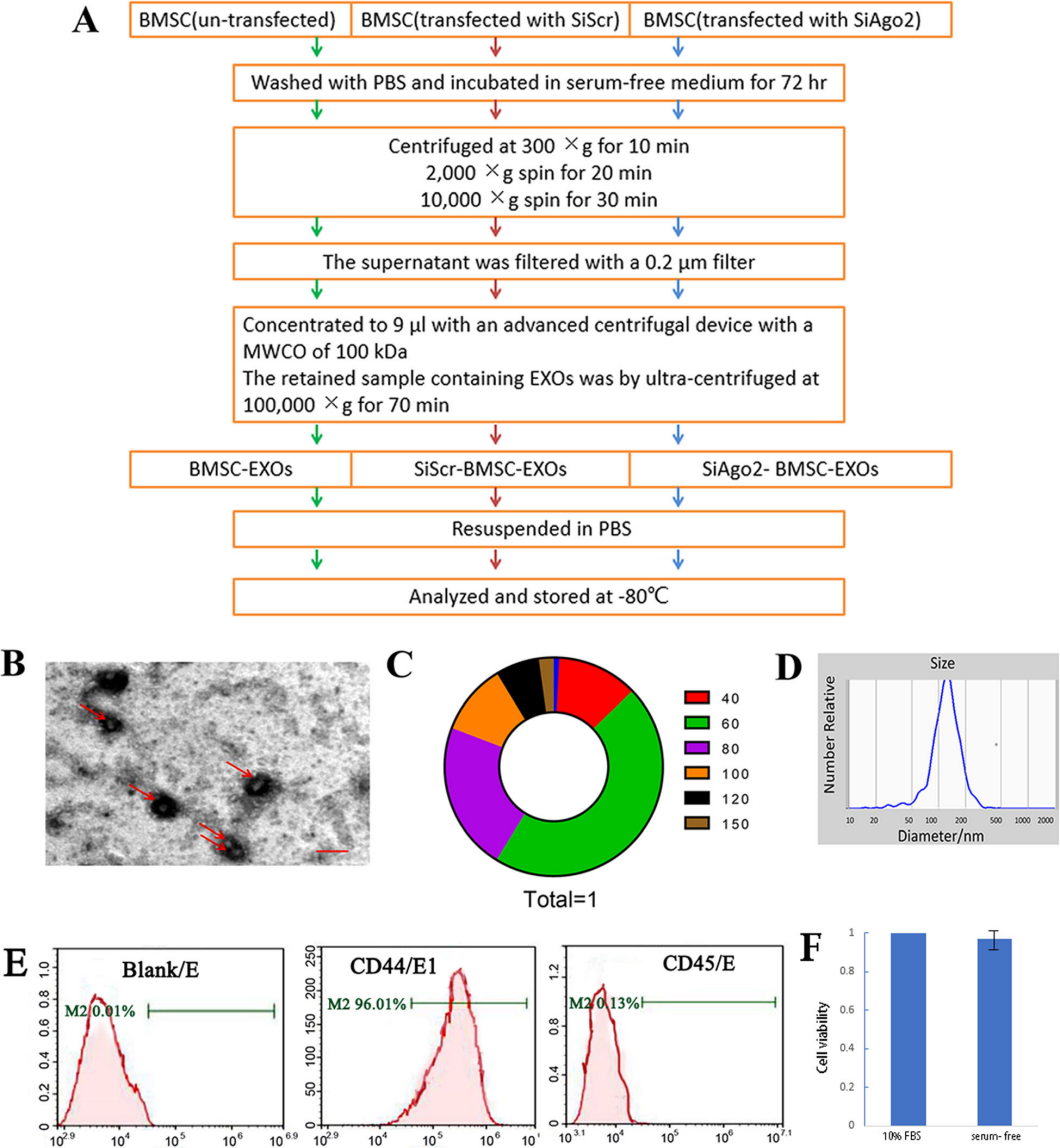
Characterization of BMSC-derived EXOs and cell viability. EXOs from the BMSC culture supernatant were assessed by TEM, NTA, and flow cytometry. **a** Protocol for EXO isolation. **b** Ultracentrifuged components of BMSC culture medium as observed by TEM. Arrows indicate particles of EXOs. **c** A schematic diagram showing the distribution of the average diameter of EXOs observed under TEM. **d** BMSC culture medium was subjected to ultra-high-speed centrifugation and detected by NTA. **e** Flow cytometry profiles of paired CD44 and CD45 marker proteins. Scale bar = 100 nm in **b**. **f** Viability measurements of BMSCs after a 72-h culture in 10% FBS and serum-free conditions. The values were normalized to present the control (10% FBS) as 100% cell viability

Passage 6 BMSCs grown to 80% confluence (transfected with SiAgo2, transfected with SiScr or nontransfected) were washed 3 times with PBS and then incubated in serum-free medium for 72 h, after which the cells were counted using a blood cell counter (approximately 2.5 × 10^7^ cells). To isolate EXOs, a well-established protocol was followed with minor modifications [29]. Briefly, the supernatant from serum-free BMSC cultures was collected and centrifuged at 300×*g* for 10 min to remove any intact cells, which was followed by centrifugation at 2000×*g* for 20 min to remove dead cells and centrifugation at 10,000×*g* for 30 min to remove cell debris. The supernatant was filtered through a 0.2-μm filter and then concentrated to approximately 9 μl with an advanced centrifugal device (Pall, MAP100C36, Shanghai, PRC) with a MWCO of 100 kDa. The retained sample containing EXOs was ultracentrifuged at 100,000×*g* for 70 min (Hitachi Optima TLX ultracentrifuge). All centrifugations were performed at 4 °C. EXO pellets were resuspended in PBS and stored at − 80 °C (Fig. 1a). EXO samples were analyzed using the Apogee A50 flow cytometry platform (A50-Micro, Apogee Flow Systems, UK).

### Western blotting

To detect the effect of SiAgo2 transfection, western blotting was performed. Briefly, BMSCs were collected and lysed to obtain protein. The protein concentration was determined by a BCA protein assay (Thermo Fisher, USA). Twenty milligrams of total protein samples were separated on 4–12% Bis-Tris protein gels at 150 V for 40 min, transferred to a polyvinylidene fluoride membrane, and blocked in 10% western blotting buffer for 30 min. The membrane was then stained with a primary anti-Ago2 antibody (Sigma-Aldrich, SAB4200085, St. Louis, USA) for 1 h, washed with TBST 3 times for 5 min each, and stained for 1 h with secondary antibody before a final wash and detection with Femto ECL. ImageJ software (NIH, Bethesda, Maryland, USA) was used to analyze the densities of the protein bands.

### The expression levels of miRNA in transfected BMSCs

To validate the effect of SiAgo2 transfection on miRNA levels, the expression of miR-21, miR-146A, and miR-17-92 in transfected BMSCs was further assessed using RT-PCR. Briefly, complementary DNA (cDNA) was generated from 1 μg of total RNA using an M-MLV Reverse Transcriptase (RT) Kit (Promega, USA) and miRNA-specific looped RT primers (Sangong, China) (Table 1). Real-time PCR was performed using an ABI PRISM® 7500 Sequence Detection System (Applied Biosystems, USA) and SYBR Green Real-Time PCR Master Mix (Toyobo, Japan). Data were analyzed using ABI PRISM® 7500 Sequence Detection System Software, Version 2.0.1 (Applied Biosystems, USA). The levels of miRNA in BMSCs were calculated in relation to levels of U6 RNA (internal control).

**Table 1:** Primers used for RT-PCR analysis of miRNA expression

miR-21	Forward	TTGGCATTAAGCCCCAGCAA
	Reverse	AGCCATGCGATGTCACGACC
miR-146A	Forward	ATATGGAAGGGTCATGAGGC
	Reverse	AGAGATGGTGCAAA GACCC
miR-17-92	Forward	AAGGCTTACATGTGTCCAATT
	Reverse	CACTTAGGGCAGTAGATGCT
U6	Forward	GCTTCGGCAGCACATATACTAAAAT
	Reverse	CGCTTCACGAATTTGCGTGTCAT

### Transmission electron microscopy (TEM)

EXO pellets collected by ultracentrifugation were resuspended in PBS, loaded onto a copper grid, and dried for 20 min at 40 °C, after which the whole preparation was stained with 3% phosphotungstic acid for 5 min. Subsequently, the samples were gently blotted dry with filter paper for 20 min at room temperature and observed by TEM (Hitachi H600, Japan). Five random fields for each grid were used to determine the average diameter of the EXOs. This process was repeated ten times.

### Zetasizer experiments

The size distribution and concentration of exosomes were measured with a Zeta View S/N 17-315 and the corresponding Zeta View 8.04.02 SP2 software (Particle Metrix, Meerbusch, Germany).

### Flow cytometry

EXOs were analyzed using flow cytometry (ACEA Biosciences Inc., NovoCyte 1040 Cytometer, San Diego, California, USA). A CD45 FITC (1:1000 dilution; 11–0461-80, eBioscience, Thermo Fisher Scientific, USA) or a rat CD44 PE (1:400 dilution; 12–0444-80, eBioscience, Thermo Fisher Scientific, NY, USA) antibody was added for 1 h at 4 °C in the dark before flow cytometric analysis. Data were analyzed using Novo Express TM software (ACEA Biosciences Inc., San Diego, California, USA).

### EXO uptake experiments and immunofluorescence

In vitro, BMSC-derived EXOs were labeled with a PKH67 Green Fluorescent Cell Linker Kit (Sigma-Aldrich, MINI67) according to the manufacturer’s protocol, with minor modifications. Specifically, EXOs were suspended in 0.5 ml of diluent C, to which 2 μl of PKH67 dye was added and incubated with the EXO solution for 5 min. To bind excess dye, 8 ml of complete culture medium (containing 15% FBS) depleted of EXOs by ultracentrifugation was added. The labeled EXOs were ultracentrifuged at 100,000×*g* for 70 min, after which the EXO pellets were washed with PBS and ultracentrifuged again. The EXO pellets were diluted in 100 μl of PBS and used for uptake experiments. Then, 0.5 ml of the EXO (approximately 5 × 10^6^ particles) suspension was added to a 1.5 ml of the DRG cell culture system (approximately 1 × 10^6^ cells) obtained from 6 to 8-week-old SD rats [30] and incubated for 48 h. The cells were fixed in 4% paraformaldehyde for 20 min at RT, washed with PBS, stained with an anti-β-tubulin III (Millipore, Mab5564, USA) antibody at 4 °C overnight, washed 3 times with 0.1 M PBS, and then incubated with donkey anti-mouse IgG conjugated to Alexa Fluor594 (Thermo Fisher Scientific, A-21203). Uptake of labeled EXOs by neurons/glial cells was visualized using confocal microscopy (Olympus FV1000, Tokyo, Japan). Z-stacks covering the entire cell volume were obtained, and three independent experiments were performed. Five visual fields were used to count labeled cells to calculate the mean transfection rate.

In vivo, SNC rats were administered 4 μl of PKH67-labeled EXOs (0.9 × 10^10^ in PBS) via gastrocnemius muscle injection (in the proximal 2/10 and 3/10 of the calf length, where the intramuscular nerve endings of the gastrocnemius muscle are densely distributed) [31]. L4–L6 DRG samples were obtained at 3 time points (1, 5, and 7 days after injection, with 4 rats for each time point). Half of these samples were sectioned into 30 μm thick slices and stained with DAPI (Boster, USA) for 1 min at RT. Then, the sections were washed 3 times with 0.1 M PBS. The remaining DRGs were stored at − 80 °C for qRT-PCR.

### Measurement of DRG neuron neurites

To assess the effect of BMSC-derived EXOs on neuritic outgrowth, BMSC-derived EXOs, SiScr-BMSCs, SiAgo2-BMSCs, SiScr-BMSC-EXOs, and SiAgo2-BMSC-EXOs were cultured with rat primary DRG neurons. Cultured neurons alone (PBS) were used as a negative control group. After 48 h of incubation, the number of processes and the neurite lengths of the neurons were recorded in five random visual fields.

### Experimental design for SNC injury and functional recovery

After intraperitoneal anesthesia, the sciatic nerve of an SD rat was exposed by separating the gluteal muscles and squeezing the nerve 5 mm under the infrapiriform foramen for 5 s with forceps (Dumont No. 5) until the nerve became translucent. The forceps were then rotated 120°, and the squeezing was repeated twice. This process ensured that the sciatic nerve axons in the transected nerve epineurium remained intact.

SNC rats were administered 4 μl of PBS, BMSCs, or EXOs via gastrocnemius muscle injection (in the proximal 2/10 and 3/10 of the calf length, where the intramuscular nerve endings of the gastrocnemius muscle are densely distributed) [31]. EXO-treated rats were re-divided into 4 subgroups (*n* = 6). PBS, BMSCs, and EXOs were administered with a Hamilton microinjector (Shanghai Gaoge, China); this procedure was repeated 3 times over 15 days (0, 7, and 14 days after SNC) [27] (Fig. 4a, b). The sciatic nerve functional index (SFI) and latency of thermal pain (LTP) measurements were recorded at 0, 7, 14, 21, and 28 days after SNC, and L4–L6 DRGs were collected 28 days after SNC [27].

### Footprint analysis

To investigate functional recovery, a footprint test was performed before SNC and at 7, 14, 21, and 28 days after SNC [3] (Fig. 4a). A homemade Plexiglass runway 1 m long, 15 cm wide, and 15 cm high was constructed. To prepare for the experiments, the runway was lined with pieces of clean white paper, and ink was brushed on both hind paws of each rat. Subsequently, the rat entered the runway from one side and exited from the other side, leaving experimental lateral foot (E) and normal foot (N) footprints for measurement. Three footprint variables were assessed: print length (PL, the distance from the heel to the toe), toe spread (TS, the distance from the first to the fifth toe line), and intertoe distance (IT, the distance from the second to the fourth toe line). These variables were substituted into the Bain formula, as follows:

SFI = − 38.3 × (EPL − NPL)/NPL + 109.5 × (ETS − NTS)/NTS + 13.3 × (EIT − NIT)/NIT − 8.8, where SFI = 0 is normal and SFI = − 100 indicates complete neurodegeneration [4].

### Measurement of LTP

To determine the hindlimb pain threshold, the paw withdrawal thermal latency (PWTL) test was performed before and 7, 14, 21, and 28 days after SNC (Fig. 4a). An RB-200 intelligent hot plate tester (Shanghai Yilian Medical Instrument Development Co., Ltd., China) was set to 55 °C and preheated for 15 min until the constant temperature indicator light became bright. The rats were numbered and placed on the plate. The latency from the appearance of the light to brisk withdrawal of the hindlimb paw was measured. To avoid tissue damage, the time did not exceed 40 s. Each measurement was repeated 3 times, with 15 min intervals between measurements.

### qRT-PCR

mRNA was extracted from the L4–L6 DRGs of the rats using an animal total RNA extraction kit (Generay Biotech, GK3015, Shanghai, China) for analysis. Briefly, 1 μg of total mRNA was reverse transcribed to cDNA using a HiScript II One Step RT-PCR Kit (Vazyme Biotech, P611-01, Nanjing, China). RT-PCR was performed using the CFX Connect Real-Time PCR System with ChamQ SYBR Color qPCR Master Mix (Vazyme Biotech, Q411-02) and unique primers (Table 2). Seven samples from each group were assayed.

**Table 2:** Primers used for RT-PCR analysis of gene expression

Akt1	Forward	GTGGCAAGATGTGTATGAG
	Reverse	CTGGCTGAGTAGGAGAAC
PMP22	Forward	TCGCGGTGCTAGTGTTGC
	Reverse	GACAGGACGCTGAAGATGACA
NGFr	Forward	CTGGGCTGATGCTGAATGC
	Reverse	TATCCGCCACTGTACTGGGTA
S100b	Forward	GGGTGACAAGCACAAGCTGAA
	Reverse	AGCGTCTCCATCACTTTGTCCA
VEGFA	Forward	GGCTCACTTCCAGAAACACG
	Reverse	GTGCTCTTGCAGAATCTAGTGG
HGF	Forward	ATTGCCCTATTTCCCGTTGT
	Reverse	TTTCAAACTAACCATCCACCCT
hGAPDH	Forward	CAATGACCCCTTCATTGACC
	Reverse	GACAAGCTTCCCGTTCTCAG

### Measurement of the number and diameter of regenerated nerve fibers

The distal sciatic nerve trunk of the SNC site (5 mm) was obtained on the 28th day after SNC and sectioned into 10 μm thick slices. After 20–30 min of staining with 1% toluidine blue, the slices were examined under a microscope (Olympus FV1000, Tokyo, Japan). Regenerated nerve fibers were counted in five random visual fields, and their diameters were analyzed using iP-win32 medical image metrology software (Molecular Devices, CA, USA). The measured data are expressed as $\overline{X}\pm S$.

### Statistical analyses

All statistical analyses were performed using SPSS 17.0 (IBM SPSS, Inc., Chicago, IL), and the data are presented as $\overline{X}\pm S$, with graphs constructed using GraphPad Prism (La Jolla, CA). SFI, LTP, and myelinated fiber count data were statistically analyzed by one-way ANOVA, and comparisons between groups were performed by the Bonferroni method. A *p* value < 0.05 indicated statistical significance.

## Results

### Characterization of BMSC-derived EXOs

EXO particles isolated from the culture supernatants of rat BMSCs had diameters that ranged between 30 and 120 nm, as shown by TEM (Fig. 1b, c). However, particles with a size of 40 to 120 nm accounted for the largest proportion (Fig. 1c). The EXOs were physically homogeneous, with a diameter distribution that peaked at 118.3 nm, as determined by nanoparticle tracking analysis (NTA) (Fig. 1d). The tetraspanin family of proteins including CD63, CD44, and CD9 are EXO markers [8, 29, 32]. CD44 expression, but not CD45 expression, was detected by flow cytometry (Fig. 1e). Overall, these results suggest that EXOs can be successfully extracted from the culture supernatant of rat BMSCs for further application.

### BMSC-derived EXOs can be transferred into neurons in vitro and in vivo and glial cells in vivo

**Fig. 2:**
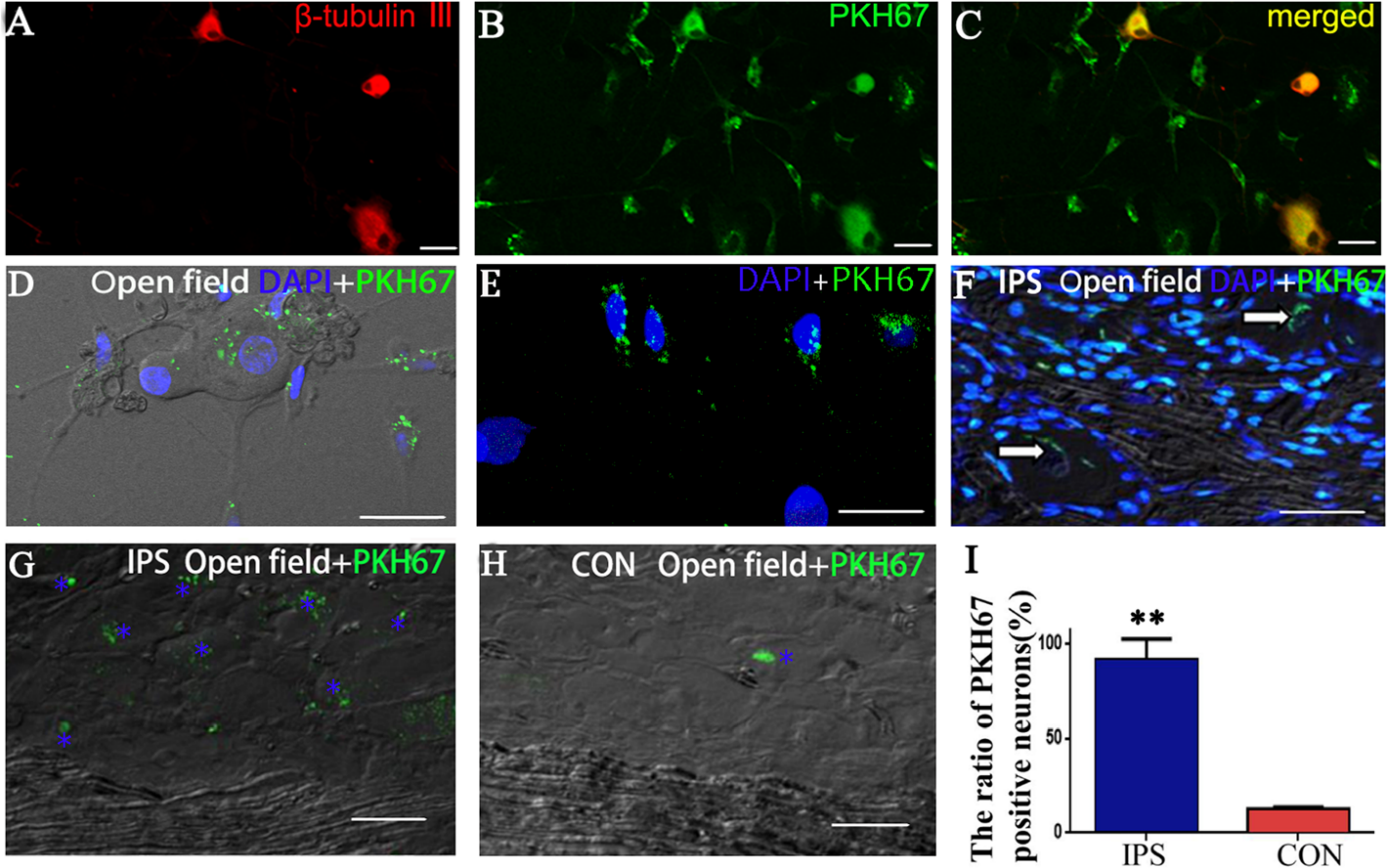
EXOs were ingested by cultured DRG neurons and glial cells in vitro and in vivo. **a**–**d** EXOs were ingested by cultured DRG neurons and glial cells after coincubation of DRG neurons (red) and EXOs labeled with PKH67 (green). **a** β-Tubulin III-labeled neurons. **b** The PKH67 marker is shown in cultured cells. **c a** and **b** merged. **d** By laser confocal microscopy, the open field shows that the cultured DRG cells were labeled with PKH67. **e** In cultured DRG cells, some of the EXOs labeled with PKH67 can be seen in the nucleus. **f**–**h** Five days after EXOs were injected into the gastrocnemius muscle, they entered DRG neuronal cell bodies and glial cells. **f** PKH67-labeled cells were visible in the ipsilateral DRG, and the PKH67 marker was present in the neuron cell body in an open field (arrow). **g**, **h** An open field under laser confocal microscopy showed the ipsilateral (**g**) and contralateral (**h**) DRG (without DAPI). Stars indicate PKF67-positive neurons in the DRG. **i** Five days after injection, the bilateral DRG PKH67-positive cell ratio (%) was statistically analyzed, and double asterisks indicate significant differences between the two sides (*p <* 0.01). Scale bar = 40 μm in **a**, **b**, and **c** and 10 μm in **d**–**h**. Images at × 40 magnification (objective) and aperture = 225 μm in **d**–**h**

To determine whether EXOs can be taken up by neurons and glial cells, BMSC-derived EXOs were labeled with PKH67 and subsequently added to a DRG culture system. After a 48-h incubation with labeled EXOs, numerous cells acquired a positive PKH67 signal (Fig. 2a–e), which indicates that BMSC-derived EXOs and their cargo can enter neurons and glial cells in vitro. PKH67 labeling was observed in neuronal nuclei by confocal laser scanning microscopy (Fig. 2e).

L4–L6 DRG sections were observed and found to exhibit PKH67 labeling by fluorescence microscopy. One day after the injection of EXOs, neurons and glia on both sides of the DRG exhibited PKH67 labeling (green). Five days after EXO injection, the number of neurons labeled with PKH67 was significantly higher in the ipsilateral (right side) DRG than in the contralateral DRG (*p <* 0.01) (Fig. 2g–i). Compared with the contralateral DRG neurons, more ipsilateral DRG neurons were labeled with PKH67 (Fig. 2g, h), and almost no labeled glial cells were found on either side. These results suggest that EXOs undergo retrograde transport into DRG neurons and glial cells by passing through blood and nerve fibers. Therefore, BMSCs can be taken up by neurons and glial cells in vitro and in vivo.

### BMSC-derived EXOs promote neuritogenesis of cultured primary DRG neurons through miRNA-dependent mechanisms

**Fig. 3:**
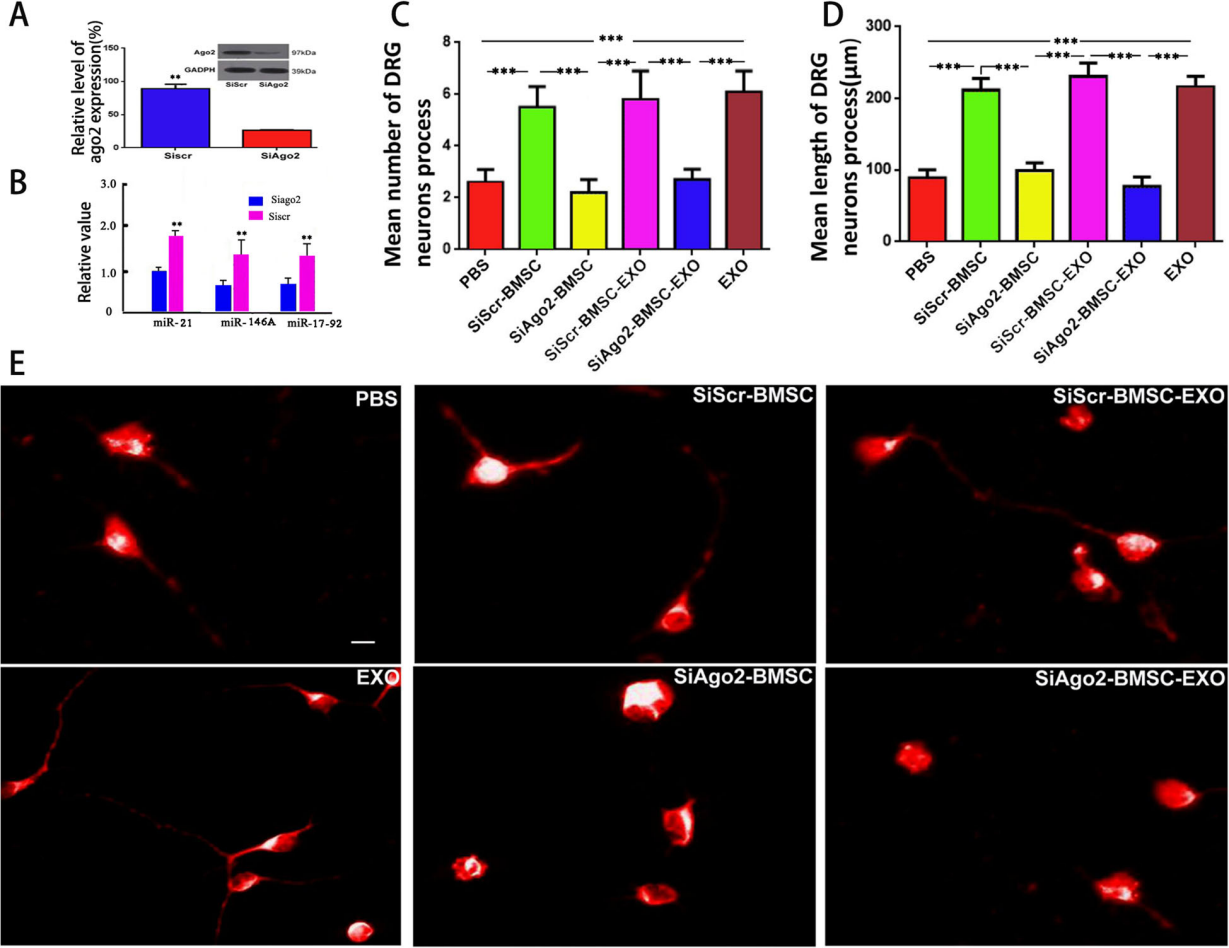
The effect of EXO miRNAs on nerve growth in vitro. Western blotting for Ago2 knockdown. **b** The relative values of miR-21, miR-146A, and miR-17-92 expression in transfected BMSCs. **c** Number of DRG neurons bearing neurites. **d** The mean length of neurites. Asterisks indicate that the difference between the two groups was statistically significant. Three asterisks indicate *p <* 0.001, and two asterisks indicate *p <* 0.01. **e** Images show the morphologies of cultured DRG neurons in each group. SiAgo2, siRNA against Argonaute-2; SiScr, scrambled siRNA control. Scale bar = 10 μm in **e**

To investigate whether the effect of EXOs on nerve growth is related to miRNA, we transfected BMSCs with an siRNA targeting Ago2 (SiAgo2) to reduce miRNA synthesis. SiAgo2-mediated Ago2 knockdown was confirmed by western blotting (Fig. 3a), and the expression of miR-21, miR-146A, and miR-17-92 in transfected BMSCs was decreased, as evidenced by RT-PCR (Fig. 3b). To quantify the effect of BMSC-derived EXOs on DRG neurons, we measured the number of processes and the neurite lengths of neurons (Fig. 3e). At 48 h after coculture, the mean number of neuronal processes in the EXO group was 6.66 ± 1.32, which was significantly higher than that in the PBS group (3.06 ± 1.43) (*p <* 0.001) (Fig. 3c, e). We detected neurite lengths of 92.6 ± 20.4 μm in the PBS group and 222.3 ± 48.8 μm in the EXO group, and this difference was statistically significant (*p <* 0.001) (Fig. 3d, e). Furthermore, we used this coculture system in which SiAgo2 was used to knock down Ago2 (SiAgo2-BMSC-EXO group) to observe the effect of EXOs secreted by BMSCs on the growth of primary cultured neurons (Fig. 3e). SiAgo2-BMSCs served as a positive control, and the SiScr-BMSC and SiScr-BMSC-EXO groups served as normal controls. After 48 h of culture, the mean neurite number (2.44 ± 0.35) and mean neurite length (66.4 ± 18.6 μm) in the SiAgo2-BMSC-EXO group were not significantly different from those in the SiAgo2-BMSC group (2.17 ± 0.54 and 75.4 ± 11.8 μm) (*p >* 0.05). However, the two average values in both groups were lower than those in the two normal control groups (5.78 ± 1.36, 231 ± 17.5 μm; 5.94 ± 1.66, 247 ± 21.87 μm), and the differences were statistically significant (*p <* 0.001) (Fig. 3c–e). These results confirm that BMSCs and their EXOs are less effective in promoting neurite outgrowth in the absence of Ago2.

### BMSC-derived EXOs promote the regeneration and functional recovery of injured peripheral nerves

#### Measurement of the number and diameter of regenerating myelinated nerve fibers

**Fig. 4:**
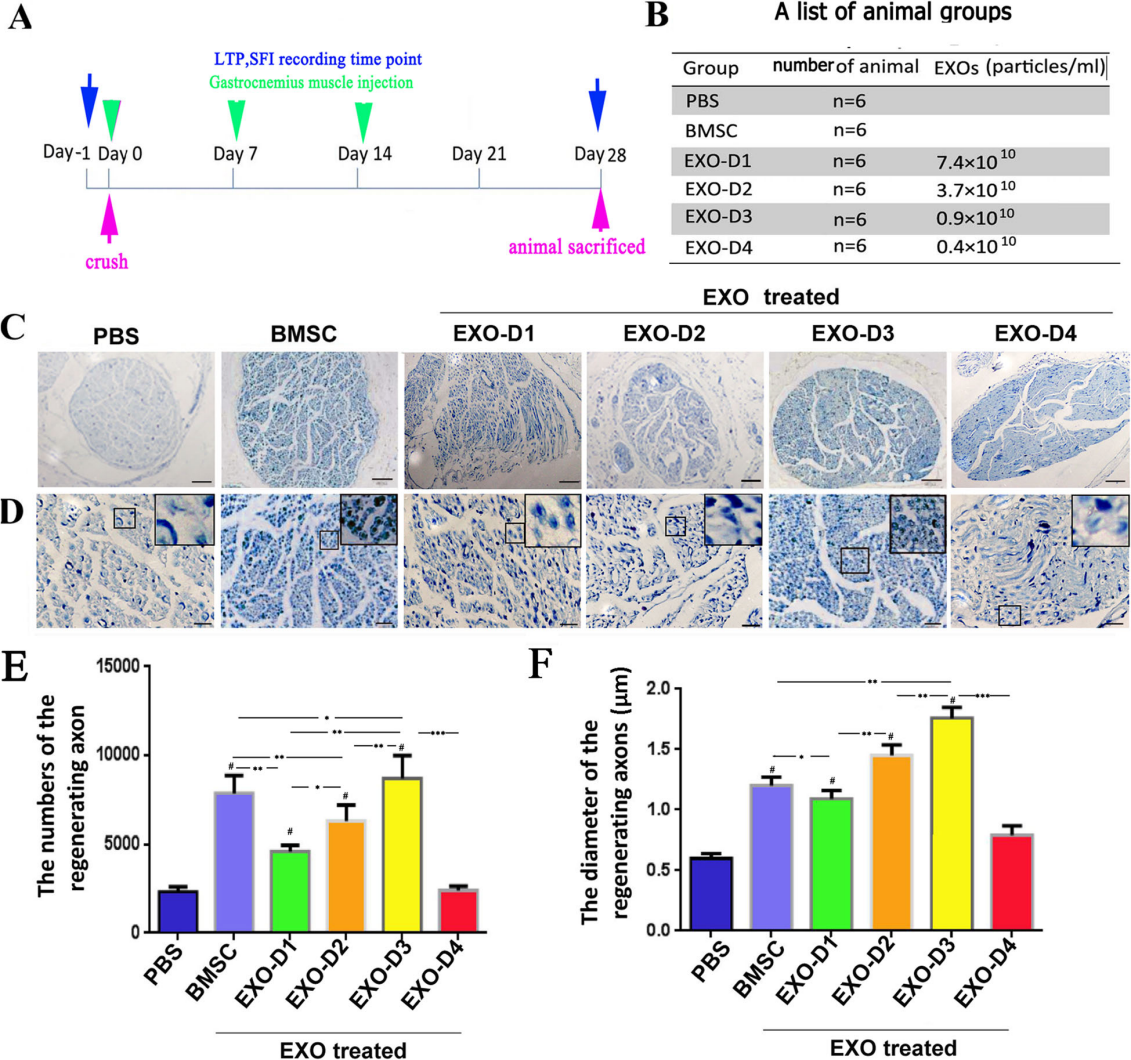
Regenerated nerve fibers were counted on the 28th day after SNC. On day 28 after surgery, the distal regenerated nerve trunk was harvested from the crush point, and transverse sections were subjected to toluidine blue staining. Regenerated fibers were observed under a microscope, and the number and diameter of regenerated myelinated fibers were statistically analyzed. **a** Schematic of the experimental design for sciatic nerve injury and functional recovery. **b** List of groups in the gastrocnemius injection experiments and the number of exosome particles injected. **c, d** Images at 100-fold or 400-fold magnification, respectively. The area in the large box was enlarged in the small box, which shows the shape of the regenerated myelinated fibers in **d**. Scale bar = 100 μm in **c**, 20 μm in **d**. **e** Statistical analysis of the number of regenerated myelinated fibers. **f** Statistical analysis of the diameters of regenerated myelinated nerve fibers. Asterisks indicate that the difference between the two groups was statistically significant. Three asterisks indicate *p* < 0.001, two asterisks indicate *p <* 0.01, and a single asterisk indicates *p <* 0.05. Number sign indicates that compared with the PBS group, *p <* 0.01, the difference was statistically significant

Nerve fibers distal to the injured site were harvested on day 28 after SNC. Toluidine blue staining was then performed on the slices. The average number of myelinated nerve fibers in the EXO-D3 group was 8100 ± 660, and significant differences were found in the EXO-D3 group compared with the BMSC group (7300 ± 460) (*p* < 0.05) and PBS group (2300 ± 260) (*p <* 0.001). The diameter of myelinated nerve fibers in the EXO-D3 group was 1.86 ± 0.23 μm, and a significant difference was found in the EXO-D3 group compared with the BMSC group (1.32 ± 0.23 μm) (*p <* 0.05). Compared with that in the PBS group (0.56 ± 0.16 μm), the diameter of myelinated nerves in the EXO-D3 group was significantly different (*p <* 0.001) (Fig. 4).

#### Measurement of the LTP

**Fig. 5:**
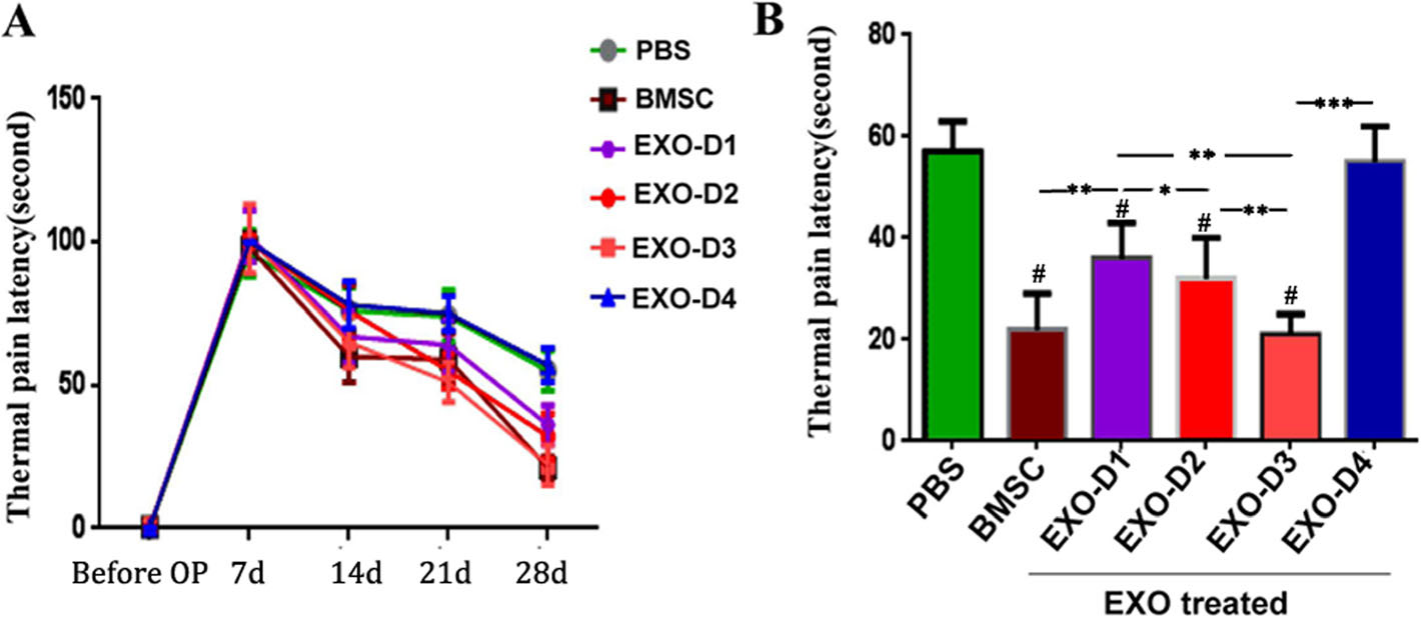
Latency of thermal pain (LTP). Preoperative and postoperative LTP at each time point. **b** On day 28 after surgery, the LTP in each group was compared and analyzed. Asterisks indicate that the difference between the two groups was statistically significant. Three asterisks indicate *p* < 0.001, two asterisks indicate *p <* 0.01, and a single asterisk indicates *p <* 0.05. Number sign indicates that compared with the PBS group, *p <* 0.01, the difference was statistically significant

Compared with that measured 1 day after surgery, the LTP had gradually decreased in each group by the 14th day after SNC (Fig. 5a). By 28 days, the LTP in the EXO-D3 group (23.61 ± 5.04 s) was superior to the BMSC group (26.8 ± 7.34 s) (*p >* 0.05) and the PBS group (58.6 ± 11.12 s) (*p <* 0.001) (Fig. 5b).

#### Gait measurement

**Fig. 6:**
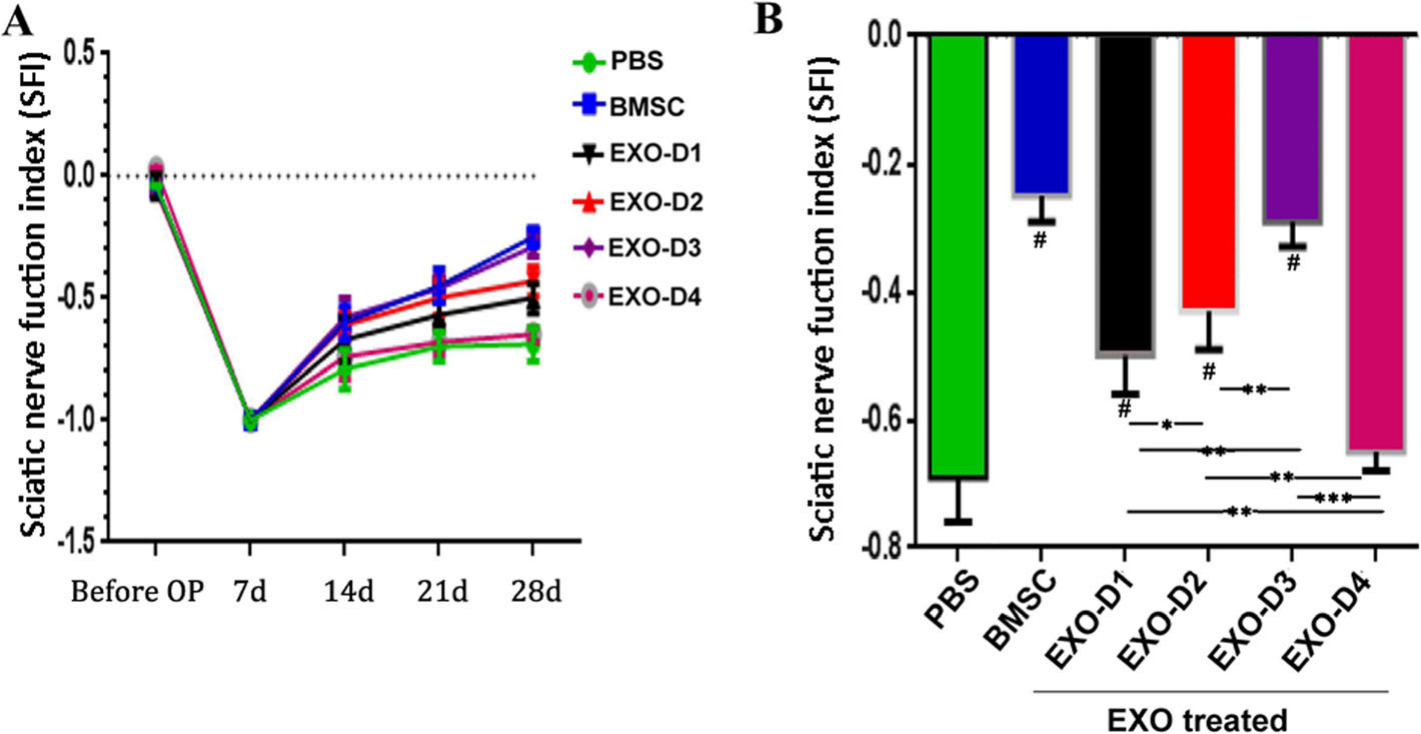
Sciatic nerve motor functional index (SFI). **a** Preoperative and postoperative SFI at each time point. **b** At 28 days after SNC, the SFI in each group was compared and analyzed. Asterisks indicate that the difference between the two groups was statistically significant. Three asterisks indicate *p <* 0.001, two asterisks indicate *p <* 0.01, and a single asterisk indicates *p <* 0.05. Number sign indicates that compared with the PBS group, *p <* 0.01, the difference was statistically significant

Furthermore, the gait of the rats was measured and analyzed, and the SFI was calculated. The SFI in each group gradually increased beginning on the 14th day after SNC (Fig. 6a). At 28 days after SNC, the SFI of the EXO-D3 group (− 0.34 ± 0.07) was not different from that of the BMSC group (− 0.28 ± 0.05) but was better than that of the PBS group (− 0.76 ± 0.09), and this difference was statistically significant (*p <* 0.001) (Fig. 6b). These results confirm that BMSC-derived EXOs accelerate regenerative fiber growth and the recovery of sensory and motor functions.

### A dose-effect relationship was observed between EXOs and nerve regeneration

#### Measurement of the number and diameter of regenerated myelinated nerve fibers

As described above, 28 days after SNC, the distal nerve trunk of the injury site was removed, and toluidine blue staining was performed. The number and diameter of regenerated myelinated fibers in the EXO-D3 group (8100 ± 660 and 1.86 ± 0.23 μm) were the highest of all the EXO-treated groups (Fig. 4c–e) and were significantly different from the values of the other dose groups (*p <* 0.01). These two values in the EXO-D2 group (6200 ± 420 and 1.49 ± 0.21 μm) were better than those in the EXO-D1 group (4810 ± 410 and 1.24 ± 0.17 μm), and those in the EXO-D1 group were better than those in the EXO-D4 group (2110 ± 215 and 0.75 ± 0.08 μm). According to pairwise comparisons, the differences were statistically significant (*p <* 0.01). However, no statistically significant difference was observed in these values between the EXO-D4 and the PBS groups (*p >* 0.05) (Fig. 4e–f).

#### Measurement of the LTP

At 28 days after SNC, the LTP of the EXO-D3 group (23.61 ± 5.04) was the shortest among the groups treated with different EXO doses, and these differences were statistically significant (*p <* 0.01). The LTP value in the EXO-D2 group (31.3 ± 6.13 s) was shorter than that in the EXO-D1 group (37.7 ± 6.00 s), and that in the EXO-D1 group was shorter than that in the EXO-D4 group (57.3 ± 11.18 s). The differences between the two groups were statistically significant (*p <* 0.01), but no statistically significant difference was found in these values between the EXO-D4 and PBS groups (*p >* 0.05) (Fig. 5b).

#### Gait measurement

At 28 days after SNC, the SFI value of the EXO-D3 group (− 0.34 ± 0.07) was superior to the corresponding values of the other EXO-treated groups. The value of the EXO-D2 group was better than that of the EXO-D1 group, and that of the EXO-D1 group was better than that of the EXO-D4 group. The differences between the two groups were statistically significant. Of all the EXO-treated groups, the SFI of the EXO-D4 group was the lowest (− 0.73 ± 0.08), and this value was not significantly different from that of the PBS group (− 0.72 ± 0.09) (*p >* 0.05). These results confirm that BMSC-derived EXOs accelerate regenerative fiber growth and sensory and motor functional recovery as a result of a specific dose-effect relationship (Fig. 6b).

### BMSC-derived EXOs upregulate PMP22, VEGFA, NGFr, and S100b gene expression in vivo

**Fig. 7:**
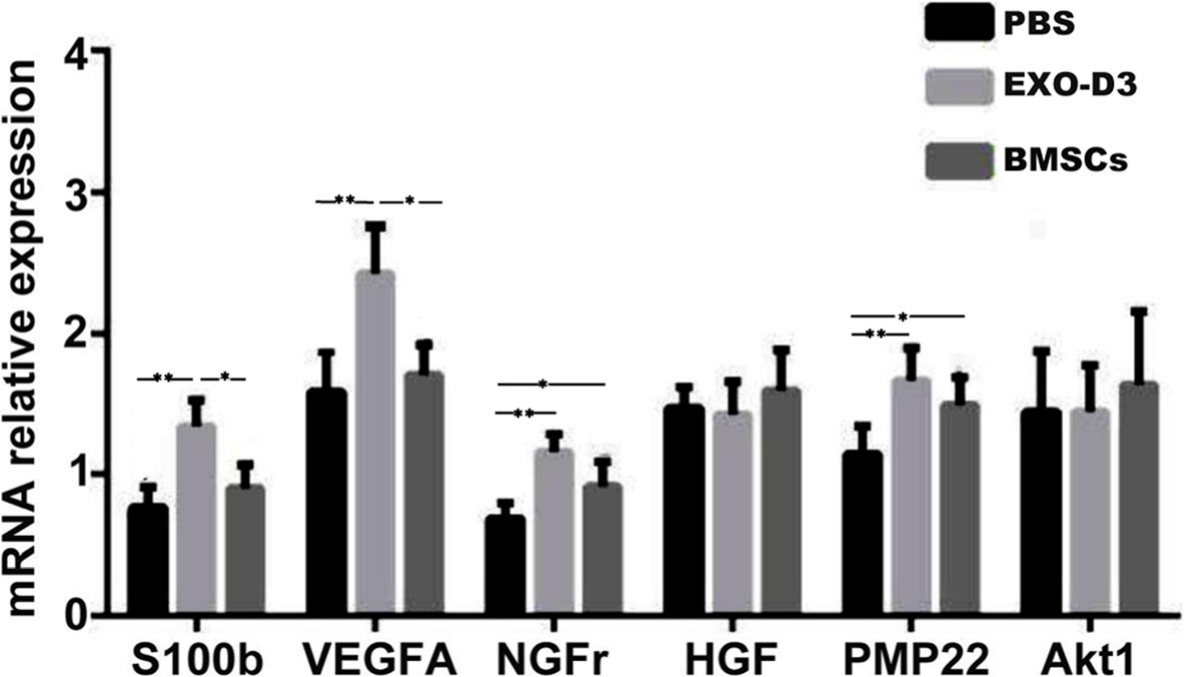
Seven days after surgery, the expression levels of genes related to DRG tissue regeneration were detected by qRT-PCR. On day 7 after surgery, the expression of regeneration-related factors in each group was compared and analyzed. Asterisks indicate that the difference in gene expression between the two groups was statistically significant: single asterisk, *p <* 0.05, and double asterisks, *p <* 0.01

To preliminarily determine whether EXO-induced nerve regeneration is related to regulation of the expression of certain factors, L4–L6 DRGs were collected from rats 7 days after SNC. The expression of PMP22, VEGFA, NGFr, S100b, Akt1, and HGF was determined by qRT-PCR. Compared with those in the PBS group, the relative expression levels of S100b, VEGFA, NGFr, and PMP22 in the EXO-D3 group were significantly higher (*p <* 0.01). Moreover, the relative expression of S100b and VEGFA in the EXO-D3 group was higher than that in the BMSC group, and the differences were statistically significant (*p* < 0.05) (Fig. 7).

## Discussion

To the best of our knowledge, this study is the first to explore the effects of different doses of EXOs on sciatic nerve regeneration by injecting BMSC-derived EXOs into the gastrocnemius muscle. To that end, we utilized an SNC model of peripheral nerve injury, which is characterized by partial injury of motor neurons in the anterior horn of the spinal cord and sensory neurons in the DRG. Since a nerve suture is not needed in this model, the artificial effect of nerve regeneration is reduced [33]. In this SNC model, we demonstrated the significant neuroprotective and axogenic effects of BMSC-derived EXOs, as well as their capacity to preserve sciatic nerve function. We also used an in vivo experiment to reveal that EXOs promote nerve regeneration and noted that the appropriate dose may increase the effect of EXOs on nerve regeneration. In addition, we preliminarily demonstrated that EXOs may promote nerve regeneration in an miRNA-dependent manner and that the upregulation of PMP22, NGFr, VEGFA, and S100b expression may play an important role in this process.

Many factors, including the regulation of gene expression in neurons, the expression of neurotrophic factors, the role of inflammatory factors at injury sites, and the activation of glial cells, affect peripheral regeneration [26]. Although it has been reported that BMSCs are not tumorigenic and have neuroprotective and axonotropic effects, the clinical application of BMSCs is limited because of the unpredictability of their long-term effects [8, 9]. Recently, EXOs released by nearly all cell types have been shown to improve the nerve regeneration process [34]. Many studies have shown that exosomes are rich in miRNAs, which can regulate various physiological cellular processes by targeting transcription factors and genes related to various cellular signaling pathways and processes, such as angiogenesis, cell transportation, apoptosis, and protein hydrolysis [35, 36]. Substantial evidence indicates that EXO miRNAs are critical vascular regeneration agents in the nervous system [37]. EXOs are also thought to play an important role in regulating nerve regeneration by transmitting miRNAs from Schwann cells, macrophages, and BMSCs [38]. By simple centrifugation [34], EXOs can be isolated relatively easily and can serve as a cell-free therapy that combines the benefits of BMSC-mediated paracrine repair without the risks [39]. EXOs can also be easily stored and do not proliferate, which means that it is easy to apply EXOs at specific doses [39, 40]. Due to their small size, EXOs can migrate into the DRG and lesion sites from the blood or nerve endings [41]. The phospholipid bilayer around EXO inclusion bodies protects them and makes them immune-inert, which is very important for therapeutic delivery [40].

Some studies have shown that EXOs are vesicles with a diameter of 40–120 nm [42], while other studies have reported the diameter of these vesicles to be 40–100 nm [43]. At present, EV isolation and purification methods cannot distinguish between different types of EVs, especially between EXOs and microbubbles less than 200 nm in diameter, or determine their cellular origins, especially in vitro. In this study, the reported EXO markers CD44 and CD63 [44, 45] were detected in these membrane vesicles, further confirming the successful isolation of BMSC-derived EXOs. The mean diameter of the EXOs detected by TEM was different from that detected by NTA. The median diameter detected by NTA (118.3 nm) was slightly larger than that detected by TEM. One of the reasons for this discrepancy is that different statistical methods were used, but it is possible that EXOs separated by ultracentrifugation contain microbubbles and broken cell components. Differential centrifugation is considered the best method to isolate EXOs from cultured cells. The first step of this process is low-speed centrifugation (300×*g* for 10 min, 2000×*g* for 10 min, 4 °C) to remove cells and fragments, and the second step is ultracentrifugation (100,000×*g* for 24 h, 4 °C) to collect EXOs [29]. Note that ultrafiltration between the two steps can be used to concentrate the EXOs and simultaneously filter out impurities and EVs larger than 220 nm in diameter. However, it remains impossible to remove microbubbles smaller than 220 nm in diameter.

EVs containing cell membranes, such as EXOs, can bind to target cells through a series of surface adhesion proteins and carrier ligands (tetramers, integrins, CD11b and CD18 receptors) and can be internalized or transfer their payload to target cells [29, 32]. At present, EXO can be labeled in many ways. Among these labels are lipophilic fluorescent labels that include PKH26 and PKH67 [46]. Here, we labeled EXOs with PKH67. Using incubation with cultured DRG cells and gastrocnemius muscle injection, we observed PKH67 labeling in the neuronal body and nucleus. We confirmed that BMSC-derived EXOs were internalized into DRG neurons and glial cells in vivo. These findings suggest that EXOs and their cargo can be transferred between these cells. Notably, we clearly observed the distribution of fluorescent markers in living neurons using open-field and fluorescence observations. This method is simpler and faster than fluorescence double-labeling.

EXOs contain both proteins and small RNAs, both of which can regulate the therapeutic effect of EXOs [47]. Ago2 knockout can reduce miRNA number and regulate miRNA function [31, 48, 49]. Mead et al. reported that RGC neuroprotection and RGC functional preservation by BMSC EXOs were significantly inhibited after Ago2 knockdown [27]. Here, we demonstrated that EXOs derived from BMSCs in which Ago2 had been knocked down attenuated the growth of neuronal processes in vitro. This protein analysis shows that BMSC EXOs carry no candidate neurotrophic factors (NTFs) as cargo but carry other proteins that can participate in the regenerative process [50, 51]. Our results suggest that miRNAs are involved in the ability of BMSC-derived EXOs to promote nerve regeneration. One of these candidate genes is miR-17-92, which is found in BMSC-derived EXOs [48]. miR-17-92 was found to target and downregulate the expression of phosphatase and tensin homolog (PTEN) [48], an important inhibitor of axonal growth and survival [52]. miR-21 has been shown to regulate the expression of PTEN [53]. miR-146A, which is expressed in the EXOs of BMSCs [54], targets epidermal growth factor receptor (EGFR) mRNA [55] and inhibits axon regeneration, while its receptor blockade promotes axon regeneration [56, 57]. BMSC-derived EXOs activate the Akt pathway [58], which is a complete pathway for neuron survival and regeneration [10].

Primary DRG culture is an accepted in vitro model in which to study peripheral nerve regeneration [59]. Neurite formation and elongation following interactions with other types of cells and/or under special culture conditions can be examined in this model [30]. Xin et al. showed that EXOs may regulate axon growth in the central nervous system [60, 61]. Our results show that the outgrowth of DRG neurons was improved after treatment with BMSC-derived EXOs for 24 h. EXOs tend to enhance axon growth and increase axon length, which is particularly important for the regeneration of peripheral nerves because the time frame for successful nerve regeneration is limited [26].

To a large extent, the lack of standardized methods for the separation, quantification, and characterization of EXOs hinders the study of EXOs. TEM is often used for the identification and morphological examination of EXOs in the early stages of EXO research and has been demonstrated to be feasible. Direct visualization by TEM is applied in the laboratory, but this method is expensive. Due to their small size, EXOs are not suitable for optical microscopy or flow cytometry [62]. When EXOs are detected by flow cytometry, a group of exosomes is usually regarded as a single EXO rather than a group of single EXOs; in other words, multiple EXOs are irradiated by the laser at the same time and often give a single signal, which leads to the underestimation of EXO concentrations [63]. NTA is a light-scattering technique designed to characterize nanoparticles [64]. In EXOs suspended in body fluid, a direct correlation is observed between the Brownian motion rate and EXO diameter. Using this established correlation, NTA can generate frequency and size distribution data by tracking the Brownian motion of EXOs in suspension, thus allowing visualization of EXOs [64]. When combined with temperature and viscosity data, NTA can estimate the size of EXOs relatively accurately with the Stokes-Einstein equation [64]. In recent years, NTA has become one of the most popular technologies used to directly count EXOs. NTA can also eliminate the cumbersome procedures involved in sample introduction, thereby reducing the possibility of contamination and more accurately estimating the EXO population count to protect the integrity of the sample and to reduce the time required to process each sample [65, 66]. The disadvantage of conventional NTA is that this technique can simultaneously measure the volume and concentration of EXOs but cannot correctly distinguish between EXO phenotypes in multidispersed samples [66]. In this study, we used microfluidic methods to measure the concentration of EXOs, but the values after repeated measurements were quite different (data not shown). After NTA measurement, the repeatability was enhanced, and the concentration was somewhat higher than that measured using microfluidic methods. Actually, this concentration may reflect the inclusion of microvesicles. We determined the number of EXOs to be injected into the gastrocnemius muscle according to the concentration of EVs measured by NTA.

In this study, a classical rat SNC model was used. Crush injury is classified as a Sunderland III degree injury [33]. Nerve trunk continuity depends on a relatively complete epineurium, and when the nerve bundle is broken, axons, the nerve intima, and the perineurium are damaged. Continuous crush injury causes less damage than discontinuous crush injury, and recovery from uninterrupted crush injury is faster, which shortens the observation period, and is not affected by repair methods. Thus, the effect of interference from the surgical repair on the functional recovery results is eliminated, and the reliability and comparability of the experiment are ensured [33].

At present, intraperitoneal injection, intravenous injection, and local application are the main methods by which EXOs are applied in vivo [67–69]. Muscle injection is primarily used for the treatment and study of skeletal muscle atrophy [70]. However, few reports have been published on gastrocnemius muscle injection of EXOs for sciatic nerve regeneration. In another study, we injected EXOs into the gastrocnemius muscle to detect markers in DRG cells, and we detected the markers in two peaks between 24 h and 5 days after injection, which indicated that EXOs reach neurons not only quickly through blood transport but also slowly through retrograde transport via nerve fibers; this increases the duration of the effect of EXOs on neurons and the activity of the EXOs [28].

Recent studies have shown that EVs are involved in multiple pathological processes, such as insulin resistance, lipid toxicity, dyslipidemia, endocrine disorders, a hypercoagulable state, and chronic inflammation [71]. In this study, although the lowest dose (EXO-D4, 0.4 × 10^10^) was not able to lead to a significant difference from the PBS group, all the other doses of EXOs promoted nerve regeneration with respect to the control group. EXOs showed a somewhat unprogressive dose effect in the EXO-D1, EXO-D2, and EXO-D3 groups, and the highest dose (EXO-D1, 7.4 × 10^10^) led to the lowest promoting effect compared with the PBS group. Regarding both the number and diameter of regenerating axons, EXO-D3 promoted an approximately 3-fold maximum increase, while EXO-D1 induced an approximate 2-fold increase (versus the PBS control).

These results reveal that EXOs show a preference for nerve regeneration but that the relationship is not “the higher dose the better”. Our findings were partly consistent with those of previous studies, which found that microvesicles inhibited neuritogenesis in cultured cortical neurons [72]. Negative neurogenic effects were reported in RGC cultures treated with high doses of EVs and were attributed to microbubbles. The filtration and removal of microbubbles from samples negated this dose-dependent effect [27]. Actually, it is almost impossible to remove all microvesicles by ultrafiltration because microvesicles are between 50 and 210 nm in diameter, and thus, some microvesicles are similar in diameter to EXOs and are difficult to remove. In addition, we cannot exclude small cellular debris, especially nuclear debris, from EXOs obtained by ultrafiltration. The presence of these microvesicles, proteins carried by nuclear debris and various RNAs, which may affect EXO function, is basically unknown [73]. These may be the reasons why EXO-D1 and EXO-D2 are much less effective than EXO-D3. In addition to the above reasons, we believe it is also possible that EXOs themselves carry a variety of active substances, such as proteins, lipids, miRNAs, dsRNAs, and cytokines. Why is EXO-D4 similar to the PBS control? One potential reason is that those active substances in EXO-D4 do not reach an effective concentration, and thus, they cannot promote nerve regeneration. Determining whether the effect of EXOs is difficult to control when EXOs are applied in large quantities is worth further assessment. Therefore, it is necessary to evaluate the safety of EXOs before clinical trials.

Toluidine blue staining of transverse nerve trunk sections remains the classic method by which the diameter of regenerated and myelinated fibers is measured [74]. In this study, toluidine blue staining of cross sections of regenerated fibers was used to measure the diameter of regenerated and myelinated fibers. Our results showed that 28 days after SNC, the number and diameter of regenerated myelinated fibers in the BMSC group and all EXO-treated groups were higher than the corresponding values in the PBS group. Interestingly, the number and diameter of regenerated fibers in the EXO-D3 group were higher than those in the other EXO-treated groups and the BMSC group. Rats in the EXO-D4 group had the fewest regenerated fibers and the smallest regenerated fiber diameter among all the EXO-treated groups. These results suggest that BMSC-derived EXOs have at least the same ability as BMSCs to promote peripheral nerve regeneration and myelin formation and that the appropriate EXO dose is crucial.

LTP is a method used to measure pain or pain response. The tail-flick and hot plate methods are commonly used to measure the LTP in animal experiments. The use of the former method is limited in animals and greatly influences the test results [75]. When radiation heat is used as a pain stimulus instead of excitation of nonpain receptors, such as skin tactile sensors and pressure sensors, the excitation stimulus of polyphasic nociceptors is simpler. During the test, the animals are relatively free to move to avoid the release of endogenous analgesic substances caused by binding and fixation [76]. This method can be used to compare the pain response of the hind limbs in the same animal, reduce individual differences between animals, and eliminate interference from human factors via the use of automatic timing, which improves the reliability and accuracy of the test results. In this study, we observed that the LTP responses in the EXO-D3 and BMSC groups were significantly shorter than those in the PBS group and showed the most extensive effect. Almost no difference was observed in LTP between the EXO-D4 group and the PBS group, but the effect in the EXO-D1 and EXO-D2 groups was not as strong as that in the EXO-D3 group. These results also suggest that BMSC-derived EXOs and BMSCs promote the recovery of sensory nerve function but that selection of an appropriate dose is important.

Walking track analysis is a comprehensive test that is widely used to evaluate motor functional recovery after traumatic peripheral nerve regeneration in rats [77]. Compared with the control treatment, a specific dose of BMSC-derived EXOs improved the motor function and speed of sciatic nerve recovery over a certain period of time; this effect was similar to that observed after the direct application of BMSCs. However, the effect of BMSC-derived EXOs at the lowest dose examined (EXO-D4 group) was not as pronounced as that in the PBS group, and the application of too many EXOs (EXO-D1 and EXO-D2 groups) did not improve motor function as effectively as a suitable dose of EXOs (EXO-D3 group). Combined with the LTP results, these findings show that BMSC-derived EXOs promote both sensory and motor nerve functional recovery.

Nerve regeneration may be related to myelin formation, glial cell activation, and angiogenesis, and studies have shown that BMSCs can synthesize various growth factors, including NGF, GDNF, NT-3, VEGFA, PMP22, HGF, and CNF [78–81]. These protein factors play an important role in peripheral nerve regeneration. Upregulation of PMP22 expression indicates that PMP22 can promote myelin formation [79]. The S100 protein, which consists of two polypeptides, S100a and S100b, is specific to peripheral nerves and is expressed at a relatively high concentration [81]. HGF plays a role in the development and maintenance of morphology in the brain and peripheral nervous system [82]. NGF plays a clear role in promoting peripheral nerve regeneration and binds to two kinds of transmembrane receptors [81]. Akt is a serine (Ser)/threonine (Thr) protein kinase that is also known as protein kinase B (PKB). At present, three subtypes of Akt have been found in mammals: Akt1, Akt2, and Akt3 [80]. Upregulation of VEGFA expression can accelerate angiogenesis and promote the recovery of nerve function. VEGFA is the most important growth factor in many cell types [83]. Our experiments showed that EXOs and BMSCs could upregulate the expression of PMP22, NGFr, VEGFA, and S100b in the DRG, which suggests that PMP22, NGFr, VEGFA, and S100b are four of the factors through which BMSCs and EXOs promote the recovery of neurological function. EXOs themselves contain not only genetic material but also a variety of proteins. Most cells, including glial cells, contain cytoskeletal proteins such as actin and tubulin in their EXOs, and these proteins play a key role in axon growth [81]. Studies have shown that active molecules such as HSP70 play an important role in neuronal metabolic support and protection [81]. Plasma galactose agglutinin-3 can affect phagocytosis of the myelin sheath. Plasma galactose agglutinin-3 has been shown to be upregulated in Schwann cells after nervous system injury and is also present in many types of microvesicles. Myelin proteins such as MAG and PLP can also be isolated from Schwann cell EXOs [84]. Studies have confirmed that AMPA, a protein found in neuronal EXOs, can be transferred between neurons [85, 86]. EXOs can pass through natural barriers, such as the blood-brain barrier and blood-nerve barrier. These results show that BMSC-derived EXOs can also promote peripheral nerve regeneration by upregulating the expression of some factors related to peripheral nerve injury, which further enriches our understanding of the regulatory function of EXOs. Therefore, we believe that BMSC-derived EXOs have the potential to replace BMSCs for the repair of peripheral nerve injury.

## Conclusions

The evidence obtained in this study suggested that BMSC-derived EXOs promote peripheral nerve regeneration and that the mechanism may involve the miRNA-mediated regulation of the expression of regeneration-related genes, such as VEGFA and S100b.

Furthermore, we used in vivo experiments to demonstrate for the first time that there is no clear positive correlation between nerve regeneration and EXO dose. High doses of EXOs do not produce the most substantial effect. We believe it is necessary to use EXOs as clinical therapeutic agents or drug carriers, improve EXO purification technology, and evaluate the preclinical safety of EXOs. The toxicity and negative effects of EXOs also deserve substantially more attention.

## Data Availability

The datasets used and/or analyzed during the current study are available from the corresponding author on reasonable request.

## Abbreviations

BMSC: Bone marrow stromal cell
EXOs: Exosomes
SNC: Sciatic nerve crush
SFI: Sciatic nerve functional index
LTP: Latency of thermal pain
DRG: Dorsal root ganglion
MSCs: Mesenchymal stem cells
EV: Extracellular vesicle
RGCs: Retinal ganglion cells
TEM: Transmission electron microscopy
NTA: Nanoparticle tracking analysis
